# Emergence and evolution of rare ST592 *bla*_NDM-1_-positive carbapenem-resistant hypervirulent *Klebsiella pneumoniae* in China

**DOI:** 10.3389/fcimb.2025.1565980

**Published:** 2025-03-31

**Authors:** Huan Zhang, Su Dong, Caiping Mao, Yuejuan Fang, Junjie Ying

**Affiliations:** ^1^ Department of Clinical Laboratory, Zhejiang Cancer Hospital, Hangzhou Institute of Medicine (HIM), Chinese Academy of Sciences, Hangzhou, Zhejiang, China; ^2^ Department of Clinical Laboratory, Shaoxing Hospital of Traditional Chinese Medicine Affiliated to Zhejiang Chinese Medical University, Shaoxing, Zhejiang, China; ^3^ Department of Pharmacy, Quzhou Maternal and Child Health Care Hospital, Quzhou, China; ^4^ Department of Urology, The Quzhou Affiliated Hospital of Wenzhou Medical University, Quzhou People’s Hospital, Quzhou, China

**Keywords:** WGS, ST592, NDM-1, evolution, CR-hvKp

## Abstract

**Objectives:**

This study aimed to characterize the genomes of two rare ST592 *Klebsiella pneumoniae* isolates and to explore their evolution into carbapenem-resistant hypervirulent *K. pneumoniae* (CR-hvKp).

**Methods:**

The minimum inhibitory concentrations (MICs) were determined using a VITEK 2 compact system. Conjugation experiments were conducted using film matings. Whole-genome sequencing (WGS) was performed using the Illumina and Nanopore platforms. The antimicrobial resistance determinants were identified using the ABRicate program in the ResFinder database. Insertion sequences were identified using ISFinder and the bacterial virulence factors identified using the Virulence Factor Database (VFDB). The K and O loci were examined using Kleborate. Multilocus sequence typing (MLST) and replicon type identification were performed by the Center for Genomic Epidemiology. Conjugation-related elements were predicted using *oriT*finder. The plasmid structure was visualized using Circos, and a possible evolutionary model was constructed using BioRender.

**Results:**

Isolates KPZM6 and KPZM16 were identified as ST592 and KL57, respectively, and were collected from the same department. The antimicrobial susceptibility testing data revealed that KPZM16 possesses an extensively drug-resistant (XDR) profile, whereas KPZM6 is a susceptible *K. pneumoniae*. The hybrid assembly showed that both KPZM6 and KPZM16 have one pLVPK-like virulence plasmid carrying the *rmpA*, *rmpA2*, and *iucABCD-iutA* gene clusters. However, strain KPZM16 harbors one IncN plasmid carrying the carbapenem resistance genes *bla*_NDM-1_, *dfrA14*, and *qnrS1*. The results of the conjugation experiments demonstrated that the plasmid could be transferred to the recipient strain. It is possible that the NDM-1-producing plasmid was transferred from KPZM6 to KPZM16 via conjugation, leading to the formation of CR-hvKp.

**Conclusions:**

This is the first study in which complete genomic characterization of the rare NDM-1-producing ST592 *K. pneumoniae* clinical isolate was performed. This study provides a possible evolutionary hypothesis for the formation of CR-hvKp via conjugation. Early detection is recommended to avoid the extensive spread of this clone.

## Introduction

Carbapenem-resistant Enterobacteriaceae (CRE) are known to cause serious nosocomial infections associated with high mortality rates, posing a global public health threat (Hala et al., 2019). Of great concern is carbapenem-resistant *Klebsiella pneumoniae* (CRKP), which causes untreatable or nearly untreatable infections, as determined by the US Centers for Disease Control and Prevention (CDC) (Gu et al., 2018). Importantly, the carbapenem resistance rates have increased with the incidence of CRKP in China, demonstrating that CRKP remains a significant multidrug-resistant pathogen, particularly in the case of the ST11–KL64 CRKP clone, which is associated with a highly resistant and virulent epidemic in China (Zhou et al., 2023; Wang et al., 2024a). Due to its resistance to the commonly used antimicrobial drugs in the clinic, the novel β-lactamase inhibitor avibactam has been developed for the treatment of CRKP strains and has been used in combination with ceftazidime in recent years, which has exhibited excellent *in vitro* activity against CRKP (Qiao et al., 2024; Zhang et al., 2024). However, the widespread use of ceftazidime–avibactam has led to several *K. pneumoniae* carbapenemase (KPC) variants (e.g., KPC-135, KPC-33, and KPC-84) showing ceftazidime–avibactam resistance (Gong et al., 2024; Shi et al., 2024; Zhang et al., 2024).

Apart from resistance in CRKP, virulence is another important issue that requires great attention (Chen et al., 2024). There are two possible evolution pathways to becoming hypervirulent and resistant, simultaneously. Hypervirulent *K. pneumoniae* (hvKp) strains that acquire carbapenem resistance plasmids could be recognized as carbapenem-resistant hypervirulent *K. pneumoniae* (CR-hvKp). On the other hand, CRKP strains can acquire pLVPK-like virulence plasmids and are known as hypervirulent carbapenem-resistant *K. pneumoniae* (hv-CRKP) (Xu et al., 2021; Tian et al., 2022b). However, the specific evolutionary mechanisms remain unclear.

In this study, two ST592 *K. pneumoniae* isolates were obtained from the same department of one hospital in China, and their complete genetic characteristics were studied. One ST592 *K. pneumoniae* isolate might be an hvKp that only carries virulence genes, such as *rmpA*, *rmpA2*, *iroBCDN*, *iucABCD*, and *iutA*. Interestingly, the other ST592 *K. pneumoniae* isolate is likely a CR-hvKp that had acquired the NDM-1-producing plasmid. To the best of our knowledge, this is the first report on a clinical ST592 *K. pneumoniae* isolate and its evolution. This information will help in the understanding of the rare genomic characteristics of CR-hvKp bacteria and in the prevention and control of their spread in healthcare settings.

## Methods

### Bacterial isolation and identification

The *K. pneumoniae* clinical isolates KPZM6 and KPZM16 were obtained from pus samples collected from the same department of a hospital in China in 2020 and 2021 (**Table 1**). The pus was inoculated into a blood agar plate and incubated overnight at 37°C. Bacterial morphology was observed on the second day. A clone suspected to be *K. pneumoniae* was further characterized. The isolates were further identified with matrix-assisted laser desorption ionization time-of-flight mass spectrometry (MALDI-TOF MS; Bruker Daltonics GmbH, Bremen, Germany) and sequencing.

**Table 1:** Basic information of the two patients.

Patient ID	Isolate	Collection date	Age	Department	Source	Diagnosis
A	KPZM6	16.05.20	51	Anorectal surgery	Pus	Anal fistula
B	KPZM16	19.12.21	63	Anorectal surgery	Pus	Perianal abscess

### Antimicrobial susceptibility testing

The minimum inhibitory concentrations (MICs) were measured using the VITEK 2 compact system: amikacin, aztreonam, gentamicin, cefotetan, cefotaxime, cefuroxime, cefazolin, ceftazidime, ciprofloxacin, ceftriaxone, cefepime, cefoxitin, piperacillin, ciprofloxacin, imipenem, and meropenem. *Escherichia coli* ATCC 25922 was used as a control strain. The results were interpreted according to the recommendations of the Clinical and Laboratory Standards Institute (CLSI) 2021 guidelines.

### Plasmid conjugation assays via film mating

To determine the transferability of *bla*_NDM-1_-positive plasmids, conjugation experiments using *E. coli* EC600 (rifampin-resistant) as the recipient strain were conducted using the film-mating method (Yang et al., 2021b; Tian et al., 2022a). Putative transconjugants were screened on Mueller–Hinton agar plates containing rifampin (150 μg/ml) and meropenem (4 μg/ml) and were further confirmed with PCR using specific primers and MALDI-TOF MS.

### Whole-genome sequencing and bioinformatics analyses

Genomic DNA was extracted using a Qiagen Minikit (Qiagen, Hilden, Germany) and the Gentra® Puregene® Yeast/Bact kit (Qiagen, Germany) and further sequenced using the Illumina and Oxford Nanopore platforms. Hybrid assembly of the short and long reads was performed using Unicycler v0.4.8 (Wick et al., 2017). Genome annotation was performed using the National Center for Biotechnology Information (NCBI) Prokaryotic Genome Annotation Pipeline (PGAP) (http://www.ncbi.nlm.nih.gov/genome/annotation_prok/) (Tatusova et al., 2016) and Prokka (Seemann, 2014). Antimicrobial resistance genes were identified using ABRicate v1.0.1 and ResFinder 4.0. Virulence factors were identified using the Virulence Factor Database (VFDB; http://www.mgc.ac.cn/VFs/) (Liu et al., 2022). The capsular polysaccharide (K locus) and lipooligosaccharide (OC locus) were analyzed using Kleborate with the command line of kleborate, ASSEMBLIES-k (Lam et al., 2021, 2022). Multilocus sequence typing (MLST) was performed and the replicon types were identified using the Center for Genomic Epidemiology website (https://genomicepidemiology.org/). The conjugation transfer elements, including the origin site of DNA transfer (*oriT*), the type IV secretion system (T4SS) region, the type IV coupling protein (T4CP), and the relaxase, were predicted using *oriT*finder (Li et al., 2018). The insertion sequences (ISs) were identified using ISfinder (Siguier et al., 2006), and the plasmid structure was visualized using Circos (Krzywinski et al., 2009). The plasmids were compared using the online tool BLAST. A possible evolutionary model was constructed using BioRender. Default parameters were used for all software packages.

### Phylogenetic analysis of the ST592 *K. pneumoniae* strains

The ST592 genomes of the *K. pneumoniae* in this study and other *K. pneumoniae* genomes from the BIGSdb-Pasteur (https://bigsdb.pasteur.fr/klebsiella/) and BacWGSTdb (http://bacdb.cn/BacWGSTdb/) databases were used to establish phylogenetic trees. Snippy v4.4.5 (https://github.com/tseemann/snippy) was utilized to align the Illumina reads against a reference (accession no. GCA_000693075.1) and to generate a core genome alignment (Zhang et al., 2022). Core SNP alignment was used to generate a maximum likelihood (ML) phylogenetic tree using RaxML v8.2.12, with the GTRGAMMA model. The generated tree files were visualized using iTOL software.

## Results

### MICs and the antimicrobial resistance gene profile

Antimicrobial susceptibility testing revealed that the clinical isolate KPZM16 possesses an extensively drug-resistant (XDR) profile: it was resistant to all of the tested cephalosporins, ciprofloxacin, imipenem, and meropenem, but remained susceptible to amikacin, aztreonam, and gentamicin. However, the strain KPZM6 was found to be carbapenem-susceptible, and the results of the assays showed that it was susceptible to all of the antimicrobial agents tested.

Analysis of the genome of KPZM16 revealed that, in addition to co-harboring chromosomal *bla*_SHV-26_, a series of genes conferring resistance to β-lactams (*bla*_NDM-1_), the strain also contains genes associated with trimethoprim/sulfamethoxazole (*dfrA14*) and quinolones (*qnrS1*) (**Table 2**). However, in the case of KPZM6, only *bla*_SHV-26_ was detected.

**Table 2:** Molecular characterization of the genomes of clinical strains KPZM6 and KPZM16.

Isolate ID	Genome	Replicon	Size (bp)	Resistance gene	Accession number
KPZM6	Chromosome	–	5,143,477	*bla*_SHV-26_	PRJNA1212823
	pKPZM6-1	IncFIB(K), IncHI1B	253,679	–	PRJNA1212823
KPZM16	Chromosome	–	5,048,454	*bla*_SHV-26_	PRJNA1213023
	pKPZM16-1	IncFIB(K), IncHI1B	208,216	–	PRJNA1213023
	pKPZM16-2	IncFIB	111,160	–	PRJNA1213023
	pKPZM16-3	IncN	59,596	*dfrA14*, *qnrS1*, *bla*_NDM-1_	PRJNA1213023
	pKPZM16-4	–	5,905	–	PRJNA1213023

– Not detected.

### Virulence factors in the two clinical strains

Numerous virulence factors were found in the clinical isolates KPZM6 and KPZM16, including the iron–enterobactin transporter-related protein gene (*fepABCDG*), the type 3 fimbriae (*mrkABCDFHIJ*), and the type 1 fimbriae (*fimABCDEFGHI*). Other important virulence factors, including *rmpA*, *rmpA2*, *iroBCDN*, *iucABCD*, and *iutA*, were further identified.

### Multilocus sequence typing and the K and O loci

Based on the *K. pneumoniae* MLST scheme, KPZM6 and KPZM16 were classified as rare ST592. Moreover, *Kleborate* was used and result showed the strain contains KL57 and O3b. In addition, the results showed that Wzi, an outer membrane protein lectin, was Wzi206.

### Chromosome and plasmid characterization analyses

The hybrid assembly showed that strain KPZM6 had a 5,143,477-bp circular chromosome with a GC content of 57.65% (**Table 2**). Moreover, a 253,679-bp pLVPK-like virulence plasmid with IncHI1B and RepB replicons was identified in strain KPZM6. In KPZM16, the plasmid pKPZM16-1, a 208,216-bp pLVPK-like virulence plasmid containing IncFIB(K)–IncHI1B replicons, was identified. In addition, three other plasmids were identified in this isolate, namely, pKPZM16-2 to pKPZM16-4, with sizes ranging from 5,905 to 111,160 bp and with GC contents ranging from 48.62% to 52.06% (**Table 2**). Importantly, pKPZM16-3 is an IncN plasmid that carries the carbapenem resistance genes *bla*_NDM-1_, *dfrA14*, and *qnrS1*.

### Genetic features of the virulence and resistance plasmids and evolutionary insights into the formation of the CR-hvKp strain

**Figure 1:**
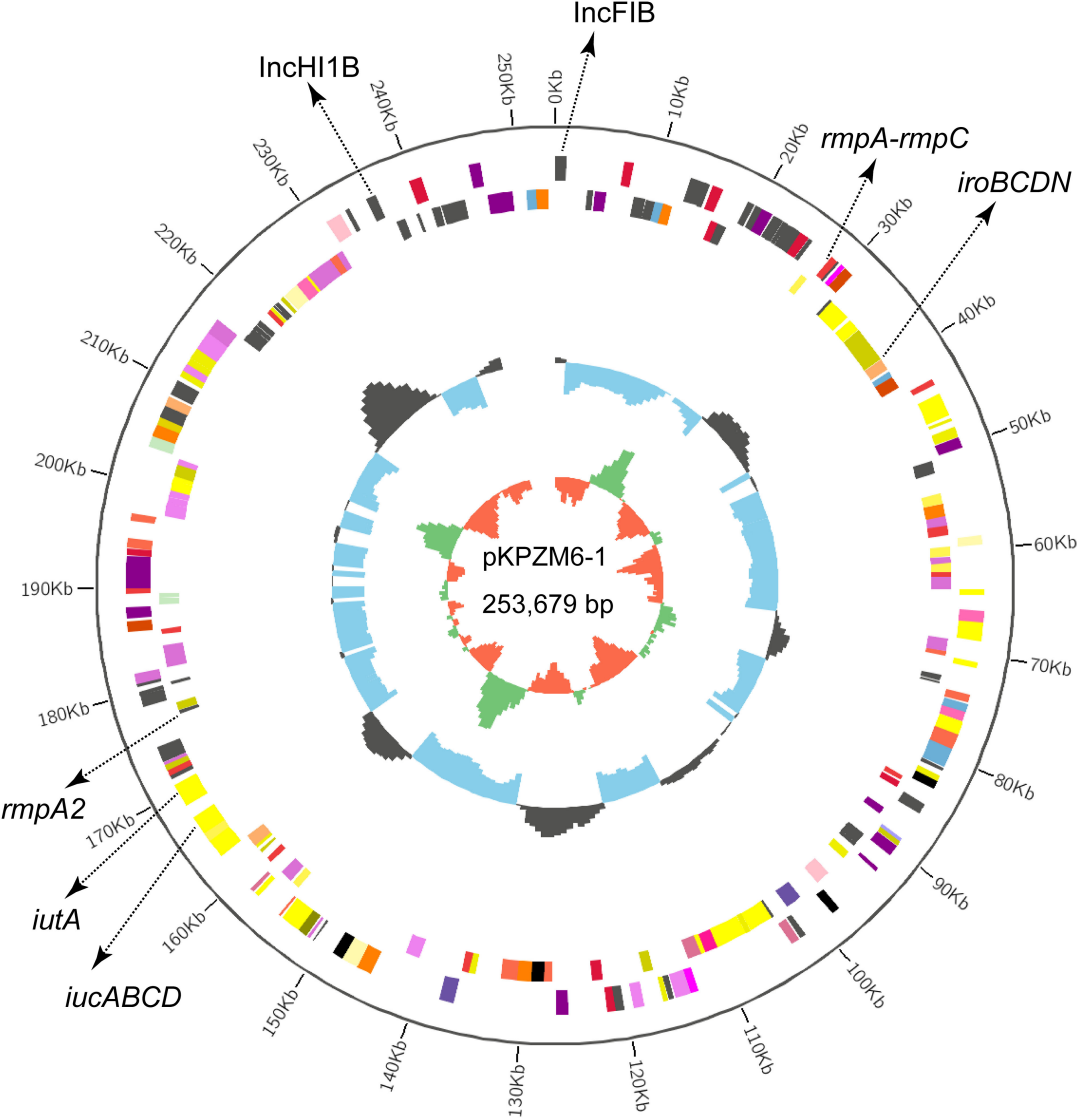
Circular map of the pLVPK-like virulence plasmid pKPZM6-1 in KPZM6. The two replicons, *rmpA*, *rmpA2*, and the *iucABCD–iutA* gene cluster are labeled.

**Figure 2:**
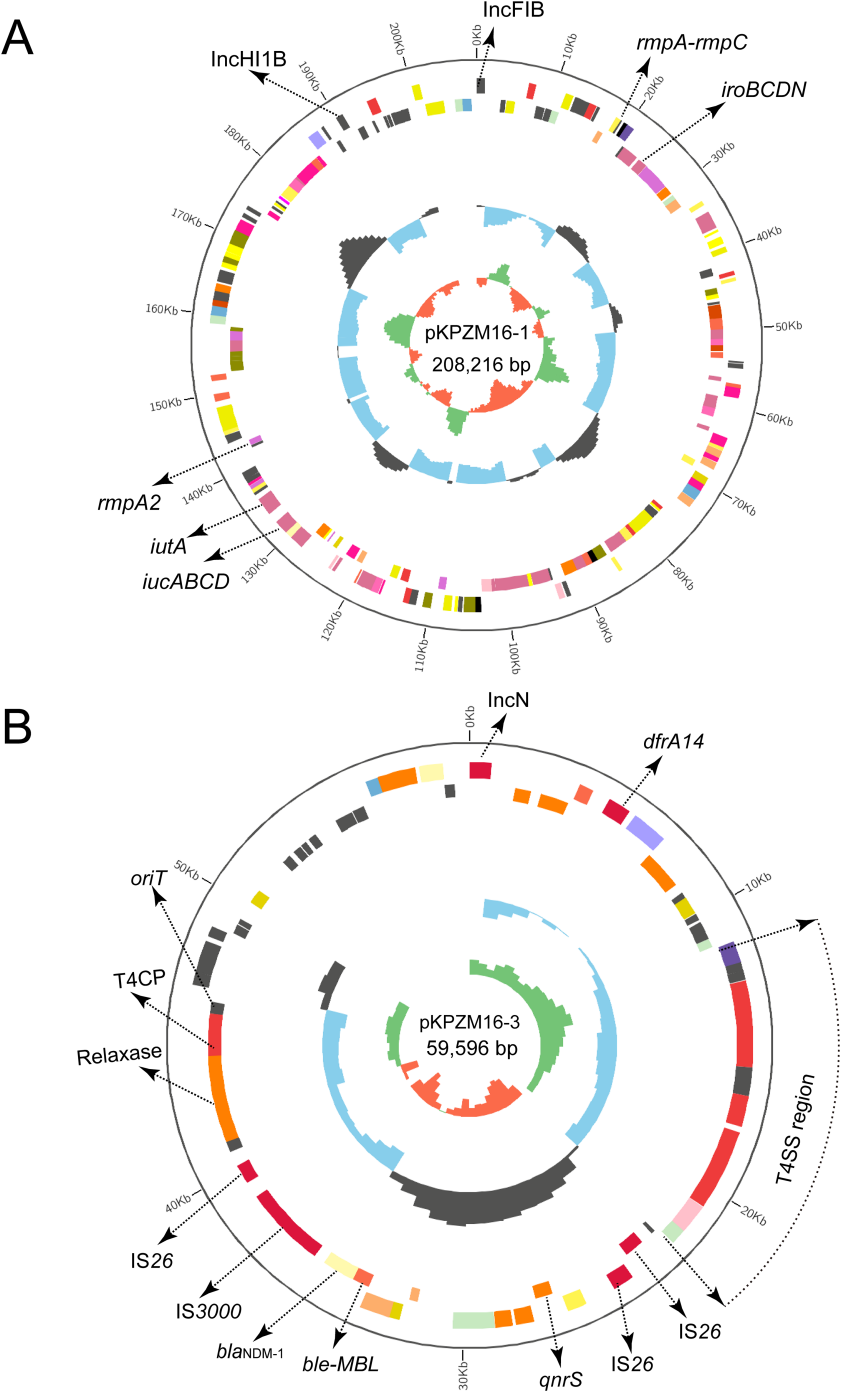
Circular map of the pLVPK-like virulence plasmid pKPZM16-1 and the *bla*_NDM-1_-positive plasmid pKPZM16-3 in KPZM16. **(A)** Circular map of the pKPZM16-1 plasmid. The two replicons, *rmpA*, *rmpA2*, and the *iucABCD–iutA* gene cluster were labelled. **(B)** Structure of the resistance plasmid pKPZM16-3. The replicon, the insertion sequences (ISs), and the resistance genes are shown. The type IV secretion system (T4SS) region, *oriT*, the type IV coupling protein (T4CP), and the relaxase are further labeled.

The basic backbones of the two pLVPK-like virulence plasmids (pKPZM6-1 and pKPZM16-1) were similar, with 77% coverage and 99.98% sequence identity. The *rmpA*, *rmpA2*, and *iucABCD-iutA* gene clusters were found in this plasmid. The plasmid structure is shown in **Figures 1** and **2A**. pKPZM16-3 is a *bla*_NDM-1_-positive plasmid (**Figure 2B**). This plasmid is composed of several resistance genes (e.g., *dfrA14*, *qnrS1*, and *bla*_NDM-1_), the *oriT*, the T4SS region, T4CP, a gene encoding a relaxase, and a series of various ISs, including IS*26* and IS*3000*. Downstream of *bla*_NDM-1_ is the bleomycin resistance-related gene *ble-MBL*. However, IS*Aba125* was not found upstream of *bla*_NDM-1_. Based on the BLASTN results using the NCBI database, the data showed similar plasmids with 100% coverage and 99.99% identity to other *K. pneumoniae* (pNDM1_LL34, accession no. CP025965.2), *Citrobacter freundii* (pNDM-Cf7308, accession no. CP092465.1), and *E. coli* (pNDM-BTR, accession no. KF534788.2) isolates. All plasmids had a size of approximately 59 kb. The results of the conjugation experiments demonstrated that it could be transferred to the recipient strain. However, both pLVPK-like virulence plasmids failed to transfer to the recipient strain.

**Figure 3:**
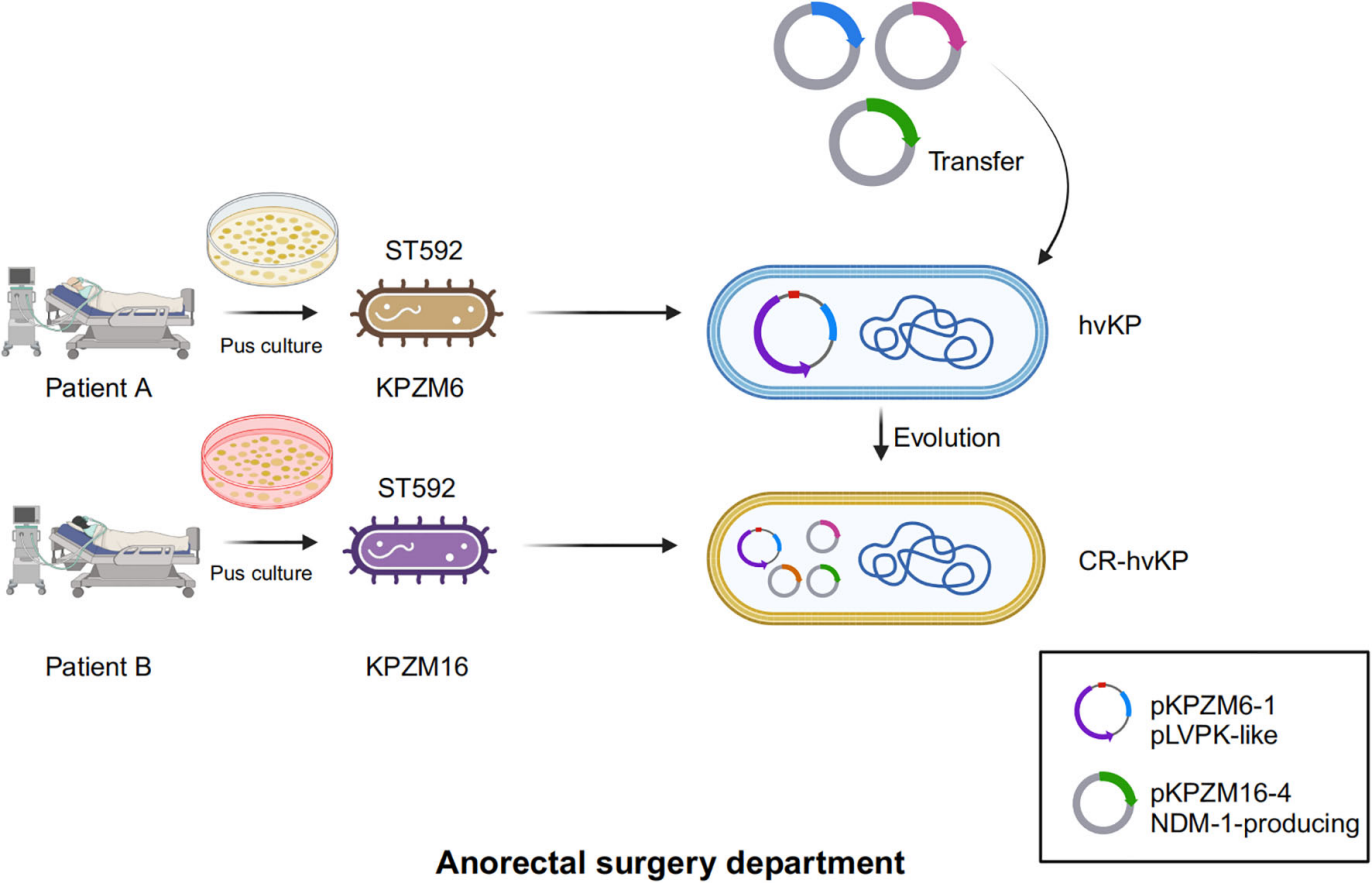
Diagram of the possible evolution mechanism of the formation of carbapenem-resistant hypervirulent *K. pneumoniae* (CR-hvKp). KPZM6 and KPZM16 were both collected from pus samples in one department. NDM-1-producing plasmid transfer from the KPZM6 (hvKp) to KPZM16 (CR-hvKp) through conjugation.

Considering that both KPZM6 and KPZM16 were collected from the same hospital department, there may have been an evolutionary process (gene flow) between them. A diagram was drawn with regard to the possible evolutionary mechanism of CR-hvKp formation based on *bla*_NDM-1_-harboring plasmid transfer (**Figure 3**). It is possible that the NDM-1-producing plasmid was transferred from KPZM6 (hvKp) to KPZM16 (CR-hvKp) through conjugation. This process may be accompanied by the co-transfer of two other plasmids (i.e., pKPZM16-2 and pKPZM16-4).

### Comparative genomics analysis of the ST592 *K. pneumoniae* isolates

**Figure 4:**
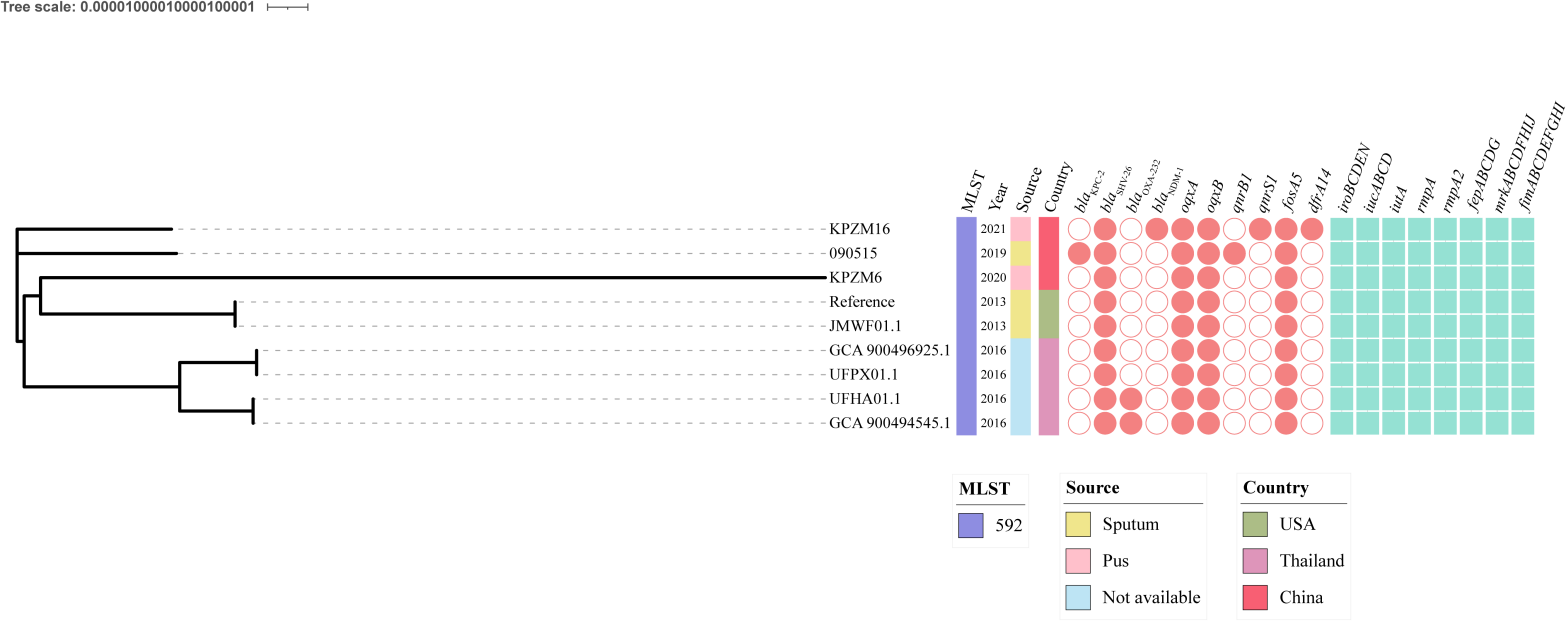
Phylogenetic analysis of ST592 *Klebsiella pneumoniae* strains. A phylogenetic tree was built using RaxML v8.2.12 under the GTRGAMMA model. The generated tree file was visualized using iTOL. The ST, sources, countries, collection year, and the resistance and virulence genes are shown.

To analyze the genetic characteristics of the ST592 *K. pneumoniae* strains from different countries, a comparative genomics analysis was performed. The data showed that nine ST592 *K. pneumoniae* strains were isolated from Thailand, China, and the USA from 2013 to 2021 (**Figure 4**). There is a huge diversity among these strains. Two strains carrying *bla*_OXA-232_, *bla*_KPC-2_, and *bla*_NDM-1_ were identified. Importantly, all ST592 *K. pneumoniae* strains carried several virulence-related genes: *iroBCDEN*, *iucABCD*, *iutA*, *rmpA*, *rmpA2*, *fepABCDG*, *mrkABCDFHIJ*, and *fimABCDEFGHI*.

## Discussion

CRKP has attracted great attention worldwide and is increasingly considered an important nosocomial pathogen in hospitals (Jin et al., 2021). In 2020, Zhou et al. reported the emergence of *rmpA*/*rmpA2*-positive ST11-KL64 isolates, which progressively replaced ST11–KL47 to become a new hypervirulent subclone, suggesting the evolution of carbapenem-resistant and hypervirulent *K. pneumoniae* (Zhou et al., 2020). However, the global occurrence of ST592 *K. pneumoniae* remains sporadic, and no NDM-1-producing ST592 CR-hvKp has been reported to date, particularly with regard to the genetic structure and evolutionary characteristics of the ST592 *K. pneumoniae* clinical strains.

Mobile genetic elements (MGEs), including the ISs, the transposons (Tn), the integrative conjugative elements (ICEs), and the integrons (In), play key roles in the horizontal acquisition of antimicrobial resistance genes in various species or between chromosomes and plasmids (He et al., 2022). The *oriT* region, the relaxase gene, the T4CP gene, and the *tra* gene cluster for the T4SS are important for plasmid transfer. In a previous study, it was shown that pLVPK-like virulence plasmids are non-conjugative owing to the lack of a complete conjugative module comprising *tra* genes, which is consistent with our findings (Yang et al., 2021a). However, in the present study, the *bla*_NDM-1_-positive plasmid was transferred to the recipient strain, as evidenced by the fact that it contains complete conjugation elements. This type of plasmid transfer contributes to the generation of CR-hvKp from hvKp, which harbors only one pLVPK-like virulence plasmid. Based on the BLASTN results from the NCBI database, the *bla*_NDM-1_-positive plasmid pKPZM16-3 shares high similarity to the pNDM1_LL34 (CP025965.2), pNDM-Cf7308 (CP092465.1), and pNDM-BTR (KF534788.2) plasmids in the *K. pneumoniae*, *C. freundii*, and *E. coli* strains, respectively, suggesting the spread of the resistance plasmid in various bacterial species. This phenomenon further explains the transfer of the *bla*_NDM-1_-positive plasmid to CR-hvKp in our study.

Several biomarkers have been used to identify CR-hvKp strains, including the presence of a combination of *rmpA* and/or *rmpA2* with *iucA*, *iroB*, or *peg-344* (Liu et al., 2020; Li et al., 2023). A study by Liu et al. suggested that the apparent changing epidemiology of hvKp is the result of ST11 classic *K. pneumoniae* (cKp) acquiring a virulence-like plasmid to replace the cKp among nosocomial infections (Liu et al., 2020). In China, the ST11 *K. pneumoniae* subclone KL64 is associated with a highly resistant and virulent epidemic, and the expansion of the ST11–KL64 clone is becoming increasingly concerning. This could be caused by multiple factors involved in mutations in antimicrobial resistance, virulence, and metabolism-associated genes (Wang et al., 2024a). Importantly, researchers have shown that the virulence plasmids in the ST11–KL64 clone are derived from a sub-lineage of ST23–KL1 (Wang et al., 2024b). However, some studies have shown that another important clone, ST23, can also transform into CR-hvKp by acquiring a *bla*_NDM_-harboring or a *bla*_KPC-2_-positive plasmid (Yan et al., 2021; Gu et al., 2025). This would also appear to be the case with the strains analyzed in the present study.

There are some limitations to this study, including the lack of data concerning the virulence level of this strain. This could be assessed using insect larval or murine models. In addition, the plasmid transfer frequency of the NDM-1-positive plasmid could be measured via conjugation. Lastly, we did not obtain direct evidence of the plasmid transfer and evolution into CR-hvKp.

## Conclusion

To our knowledge, this is the first study in which the complete genomic characteristics of rare ST592 *K. pneumoniae* clinical isolates have been investigated. This study provides a possible evolutionary hypothesis for the formation of CR-hvKp via conjugation. Considering the huge threat posed by CR-hvKp bacteria, which are prevalent in the pus of patients in healthcare settings, early detection and control should be strengthened to avoid the wide dissemination of this high-risk clone.

## Data Availability

Publicly available datasets were analyzed in this study. This data can be found here: Bioproject PRJNA1212823 and PRJNA1213023.
